# A rodent model of anterior ischemic optic neuropathy (AION) based on laser photoactivation of verteporfin

**DOI:** 10.1186/s12886-018-0937-5

**Published:** 2018-11-22

**Authors:** Jing-yu Min, Yanan Lv, Lei Mao, Yuan-yuan Gong, Qing Gu, Fang Wei

**Affiliations:** 1 Department of Ophthalmology, Shanghai General Hospital, Shanghai Jiao Tong University School of Medicine, NO.100, Haining Road, Hongkou District, Shanghai, 200080 China; 2 Shanghai Key Laboratory of Ocular Fundus Diseases, NO.100, Haining Road, Hongkou District, Shanghai, 200080 China; 3 Shanghai Engineering Center for Visual Science and Photomedicine, NO.100, Haining Road, Hongkou District, Shanghai, 200080 China

**Keywords:** Anterior ischemic optic neuropathy, Verteporfin, Laser photoactivation, RGCs

## Abstract

**Background:**

A rodent model of photodynamic AION resulting from intravenous verteporfin is presented. The analysis of the morphological function, the pathological changes and the potential mechanism of action were further investigated.

**Methods:**

Photodynamic treatment was conducted on the optic nerve head (ONH) following administration of the photosensitizer. The fellow eye was considered as sham control. Fundus Fluorescein angiography (FFA), spectral domain optical coherence tomography (SD-OCT) and Flash-visual evoked potential (F-VEP) recordings were conducted at different time points. Immunohistochemistry was used to observe apoptotic cell death (TUNEL) and macrophage infiltration (ED-1/Iba-1). Retrograde labeling of retinal ganglion cells (RGCs) was used to evaluate the loss of RGCs.

**Results:**

After laser treatment, SD-OCT indicated optic nerve edema, while FFA indicated late leakage of the ONH. F-VEPs were distinctly reduced compared to control eyes. The number of apoptotic RGCs peaked on day 14 (5.71 ± 0.76, *p* < 0.01). The infiltration of ED-1 and Iba-1 increased on the 3rd day following PDT, while it peaked on day 14 (67.5 ± 9.57 and 77.5 ± 12.58 respectively, *p* < 0.01). Following 3 weeks of AION, the densities of RGCs in the central retinas of the normal and AION eyes were 3075 ± 298/mm^2^ and 2078 ± 141/mm^2^ (*p* < 0.01), respectively.

**Conclusions:**

Verteporfin photodynamic treatment on rodents ONH can lead to functional, histological, and pathological changes. This type of animal model of AION is easy to establish and stable. It can be used for studying the mechanism and neuroprotective medicine of AION injury.

## Introduction

Anterior ischemic optic neuropathy (AION) is the leading cause of sudden optic nerve-related (ON-related) vision loss in elderly people. Approximately 11.7% of these cases experience central vision abnormalities [1]. In addition, AION was notably noted in one eye, according to a previous study conducted in China, with an incidence rate of 0.03+/− 0.03% (mean +/− standard error) per 5 years (1:16,000 subjects annually) [2].

Based on clinical studies, AION is considered a secondary symptom to ischemia, which is predominantly caused by the posterior ciliary arteries [3], although the exact pathophysiology that leads to axonal degeneration remains undiscovered. In addition, there are a lot of risks, scuh as diabetes mellitus, hypertension, hypercholesterolemia, and crowded structure of the optic disc with small cup to disc ratio. To date no effective method has been reported to prevent vision loss following the development of AION. Thus, it is necessary to establish an animal model of AION that can be used to assess the potential benefits of neuroprotective strategies.

Bernstein et al. conducted the first study that used a photodynamic model of AION in rats by photoactivating rose Bengal with a 532 nm laser [4]. Since then, this model had been further investigated by several neuroprotective agents [5–8]. Differences had been noted among the severity of this disease in rodents due to a variety of factors, such as the differences of operation and individuals worked, and the very short half-life of rose Bengal, which required a rapid operation of photoactivation following administration. Furthermore, the photosensitizing agent mesoporphyrin IX dihydrochloride was used in establishing the model of AION via intraperitoneal injection [9], although this type of model had not been widely used due to its short time of discovery.

Verteporfin is a benzoporphyrin derivative and is proposed for treating several eye diseases, such as choroidal neovascularization (CNV), pathological myopia, and/or polypoidal choroidal vasculopathy (PCV). Photodynamic treatment following intravenous injection of verteporfin in rodents can lead to edema of the normal choroid and retina [10]. Hence this type of photosensitizer was used in the development of the AION model. Furthermore, verteporfin exhibits a longer half-life than rose Bengal, whereas the photosensitizing agents that were used in the present study were recycled by the discarded parts used for the clinical procedures required for AION examination. Consequently, verteporfin was selected to construct the AION model in the current study.

In the present study, we describe an alternative approach to induce AION inrodents. Furthermore, we studied the morphology, function, and the mechanism of action of this model.

## Method

### Animals

All animal experiments adhered to the ARVO statement for the Use of Animals in Ophthalmic and Vision Research. Adult male Sprague Dawley (S-D) rats weighing 180–200 g were purchased from the Shanghai Laboratory Animal Center of the Chinese Academy of Sciences and were used for all the experiments. All animals were housed in cages at constant temperature, fed with a standard diet ad libitum and maintained under a 12-h light/12-h dark photoperiod. All types of surgery and manipulation were conducted in the Shanghai Key Laboratory of Fundus Disease. All procedures were carried out under sedation. Sedation was achieved by intraperitoneal injection of 10% (*w*/*v*) chloral hydrate(3.5 ml/kg). The pupils of anesthetized rats were dilated with one drop of 5% tropicamide, and the corneal was anesthetized with one drop of 0.4% oxybuprocaine hydrochloride. The number of animals used for the morphological and functional analyses of each group was *N* = 6.

### Induction of AION

A total of 6 mg/m^2^ of verteporfin (Novartis Ophthalmics Europe Ltd., Basel, Switzerland) was injected intravenously through the tail vein in order to induce optic nerve head ischemia. Following administration of the photosensitizer for 1 to 10 min duration, the laser was applied on the ONH of the left eye of each animal. The optic nerve head was subjected to a laser beam at a wavelength of 689 nm across a 500-μm diameter spot size for 158 consecutive secs. The laser energy used was 600 mW/cm^2^. The laser beam was used for the fellow eye at the ONH with no laser emission and the same operation parameters as stated above.

### Fluorescein angiography and optical coherence tomography

Fundus fluorescein angiography (FFA), and spectral domain optical coherence tomography (SD-OCT) were conducted on days 0, 1, 3 and 7 following treatment to observe the progress of the optic nerve head edema and the related retinal response. A solution of 10% Fluorescein sodium (Alcon Laboratories Inc. Switzerland) was injected intraperitoneally. The angiographs were recorded following the change in coloration of the conjunctiva that appeared yellow in color (Heidelberg Engineering, Heidelberg,Germany). Retinal structure and retinal thickness were measured using SD-OCT (Heidelberg Engineering). Retinal scans were centered on the optic disc in both control and injured eyes.

### Flash-visual evoked potentials (F-VEPs)

The F-VEP was conducted on days 1, 7, 14 and 21 following treatment with a Ganzfeld system (RetiPort, Roland Consult, Brandenburg, Germany). All animals had their pupils dilated and were anesthetized. Upon examination of one of the two eyes of each animal, the contralateral eye was covered. The settings of F-VEPs were based on previous reports [6, 11], including no background illumination, a flash intensity of Ganzfeld 0 dB, a single flash with a flash rate of 1.9 Hz and a flash intensity of 3 cd.s/m^2^. The average test was conducted at 80 sweeps, whereas the threshold for rejecting artifacts was set at 50 mV and a sample rate of 2,000 Hz was used. The amplitudes of P1 for each F-VEP wave within the initial 100-ms interval were determined and used for the amplitude analysis (amplitude of P_1_ = amplitude of P_1_- amplitude of N_2_) [6, 12].

### Immunohistochemistry

Animals were euthanized on days 1, 4, 7, 14 and 21 following laser application. The eyes were enucleated, fixed in 4% paraformaldehyde (PFA) in PBS for 24 h and the anterior segment was removed. Subsequently, certain eyes were dehydrated in 30% sucrose overnight and embedded in Tissue-Tek O.C.T compound (Sakura, Torrance, CA) Cross sections of 10-μm in diameter were performed. The sections were incubated with mouse anti-CD68 monoclonal antibody(Serotec Ltd., Oxford, UK) at a 1:100 dilution and/or rabbit anti-Iba-1 monoclonal antibody (Abcam Inc. Cambridge, MA) at a 1:100 dilution, at 4 °C overnight in order to identify macrophages and microglia. FITC conjugated goat anti-mouse IgG and/or FITC conjugated goat anti-rabbit IgG (Jackson Immunoresearch, West Point, PA) were incubated with the sections for 1 h. Finally, sections were counterstained with 4′,6-diamidino-2-phenylindole (DAPI) nuclear stain.

The remaining eyes that were not examined were dehydrated with a posterior eyecup and embedded in paraffin. Retinal cross sections (5 mm thick) were then cut and stained with hematoxylin and eosin (Sigma, MO,USA). The sections were photographed and measured at approximately 2 to 3 disc diameters from the optic nerve using a microscope (Olympus BX53; Olympus, Tokyo, Japan). The thickness of the retinal tissues was determined by cell counts over a distance scale of 200 mm. The retinal thickness and cell number were calculated as the mean values of at least 3 measurements in adjacent sections [13].

### TdT-mediated dUTP Nick-end labeling (TUNEL) assay

On days 1, 4, 7 and/or 14 following laser application, paraffin-embedded retinal tissue sections were deparaffinized, rehydrated, fixed with 4% PFA for 15 min at 4 °C and then subjected to enzymatic digestion with 20 mg/ml proteinase K for 8 to 10 min at room temperature. Induction of apoptosis was examined by TUNEL assay using a DeadEnd™ Fluorometric TUNEL System, according to the manufacturer’s instructions. 4′,6-diamidino-2-phenylindole (DAPI) was used to stain the nuclear regions of the tissues. TUNEL-positive cells were examined using a laser scanning confocal microscope (Zeiss LSM 510, Carl Zeiss,Germany) in vitro in 6 random fields (at least 100 DAPI-positive cells per field) for each experimental group. The level of apoptosis was expressed as the ratio of the number of TUNEL-positive cells to that of DAPI-positive cells [13].

### Retrograde labeling of RGCs with FluoroGold and morphometry of the RGCs

Following deep anesthesia, the rat heads were fixed in a stereotactic apparatus (Stoelting Kiel, Germany) and the skin covering the skull of the rats was incised. Fluoro-Gold (FG;Biotium, Hayward, CA, USA) was injected (2 μl of 4% FG in distilled H_2_O) into the superior colliculus (SC) on each side using a microsyringe, and was retained for 10 min. The animals were maintained for 1 week post-labeling and subsequently the eyes were enucleated and fixed with 4% PFA for 1 h. The retinas were examined with an Olympus BX53 fluorescence microscope (Olympus, Tokyo, Japan) with UV excitation (excitation filter, 350–400 nm; barrier filter, 515 nm) and a digital imaging system. The RGCs were examined by division into 4 quadrants (superior, inferior, nasal,and temporal), which were further divided into central (0.8–1.2 mm from the optic disc), middle (1.8–2.2 mm from the optic disc), and peripheral regions (0.8–1.2 mm from the retinal border). A total of 2 standard square areas (200 × 200 μm^2^) were measured in each region. The density of RGCs in each group of rats was expressed as the number of labeled RGCs/mm^2^ compared with the counted retinal area [14].

### Statistical analysis

The quantitative data were presented as mean ± SD. Statistical analyses were conducted using SPSS version 20 (SPSS, IL, USA). An unpaired Student’s t-test for two-group data and one-way analysis of variance followed by a post hoc Bonferroni’s multiple comparison test for three groups or more. A *p* value of lower than 0.05 (*p* < 0.05) was considered statistically significant. Each experiment was conducted three times.

## Results

### Morphology of ONH following AION induction

**Fig. 1:**
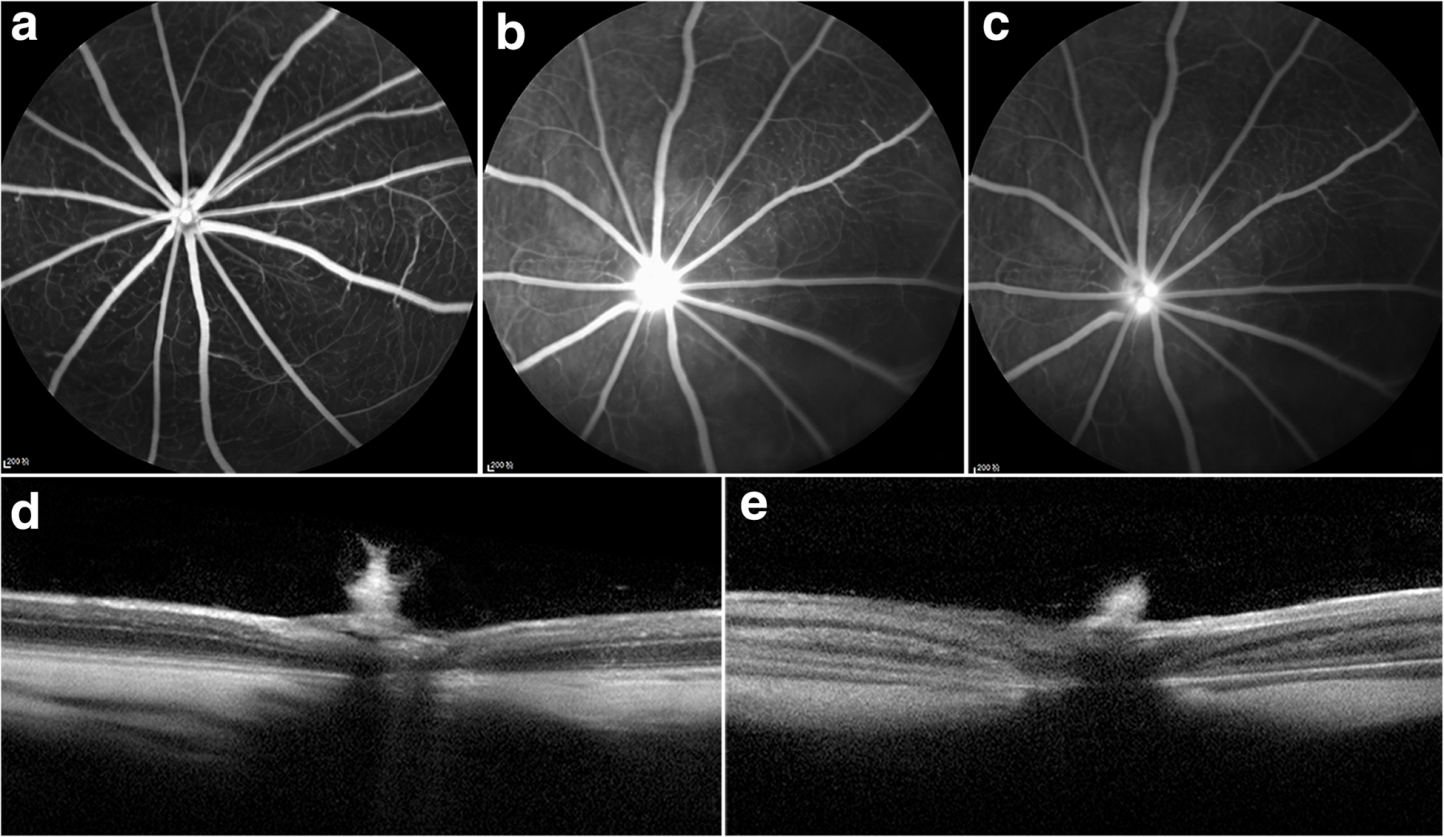
Fundus fluorescein angiography (FFA) and spectral domain optical coherence tomography (SD-OCT). **a**, **d** The control fellow eye had no abnormal changes. **b** In the mid-phase, hyperfluorescence can be dectected at the ONH in PDT eye at 1 day post-AION. **c** In the late-phase, subdued fluorescence at ONH in the same PDT eye. **e** SD-OCT showed the swelling of the retina tissue of treated eyes at 3 days post-AION

The control eye had no abnormal changes (Fig. 1a, d). In the photodynamically treated eyes, FFA indicated fluorescein leakage from the ONH vasculature on day 1, (Fig. 1b, c) which was consistent with the swelling of the retina tissue as demonstrated by the SD-OCT images. On day 3, the optic nerve edema was more pronounced (Fig. 1e). However, a resolution of the edema was noted on day 7.

### Flash-visual evoked potentials (F-VEPs)

**Fig. 2:**
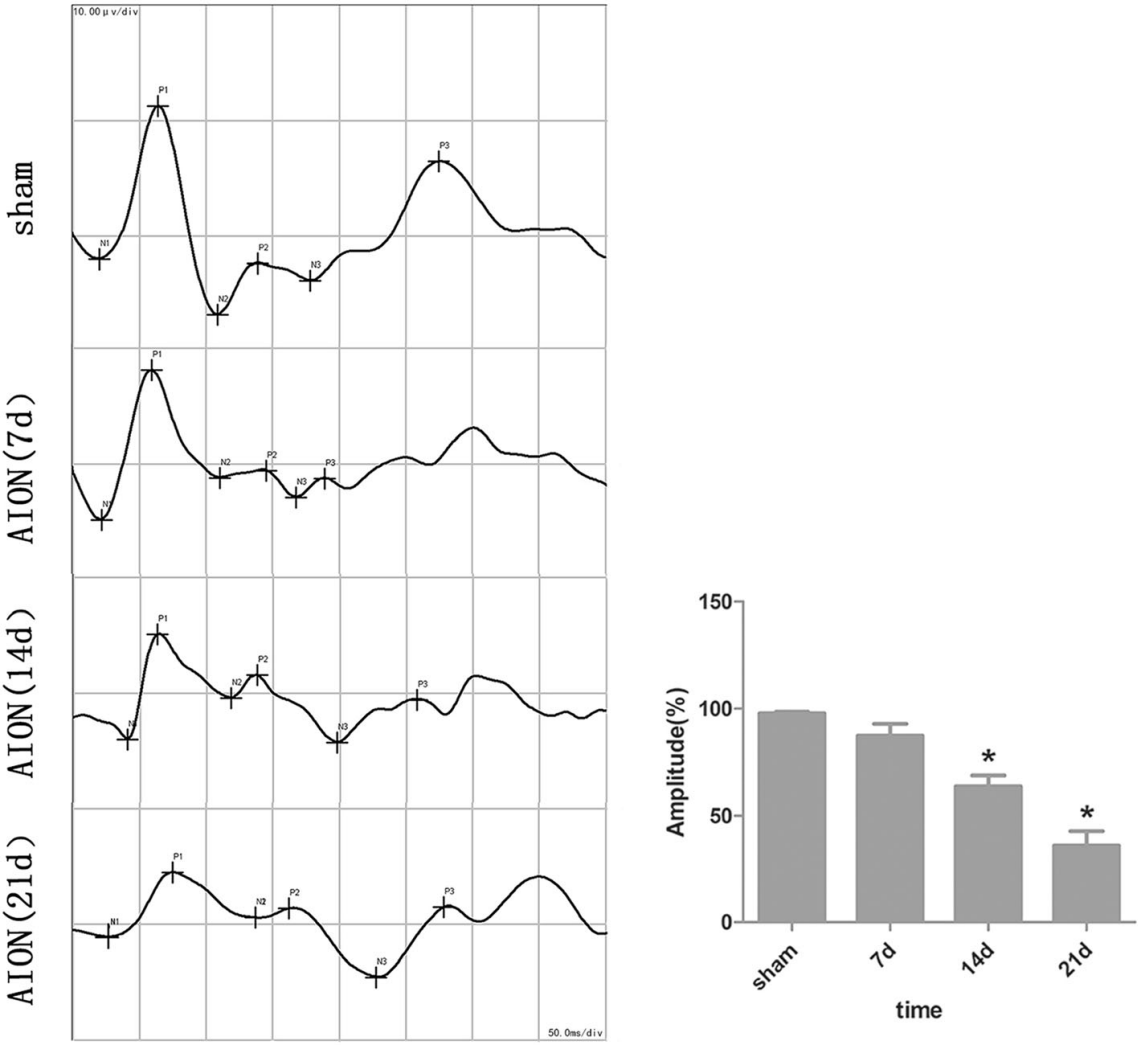
Flash-Visual Evoked Potentials (F-VEPs) in AION. F-VEP amplitudes of P1 in treated eyes measured 87.3 ± 11% of the fellow control eyes at 1 week, 67.6 ± 11.5% at 2 week, and 35.9 ± 13.6% at 3 week. We did not compare F-VEP latencies in the two data sets because of the indistinctive statistics. (*P < 0.05 in the 14d and 21d groups compared with the sham group, *N* = 6 in each group)

F-VEPs were recorded at the first, second and third week, following AION induction in order to evaluate the function of ON (Fig. 2). The F-VEPs of the treated eyes were compared with the fellow eyes of each animal in order to eliminate the variations in the F-VEP amplitude within the animal groups [15]. F-VEP amplitudes in the treated eyes (*N* = 6) were estimated to 87.3 ± 11%, 67.6 ± 11.5% and 35.9 ± 13.6% of the fellow control eyes at the first, second and third week, respectively (*P* < 0.05). F-VEP latencies in the two data sets exhibited no statistical significance, as demonstrated in previous experiments [15].

### Induction of apoptosis and death in the RGC layer

**Fig. 3:**
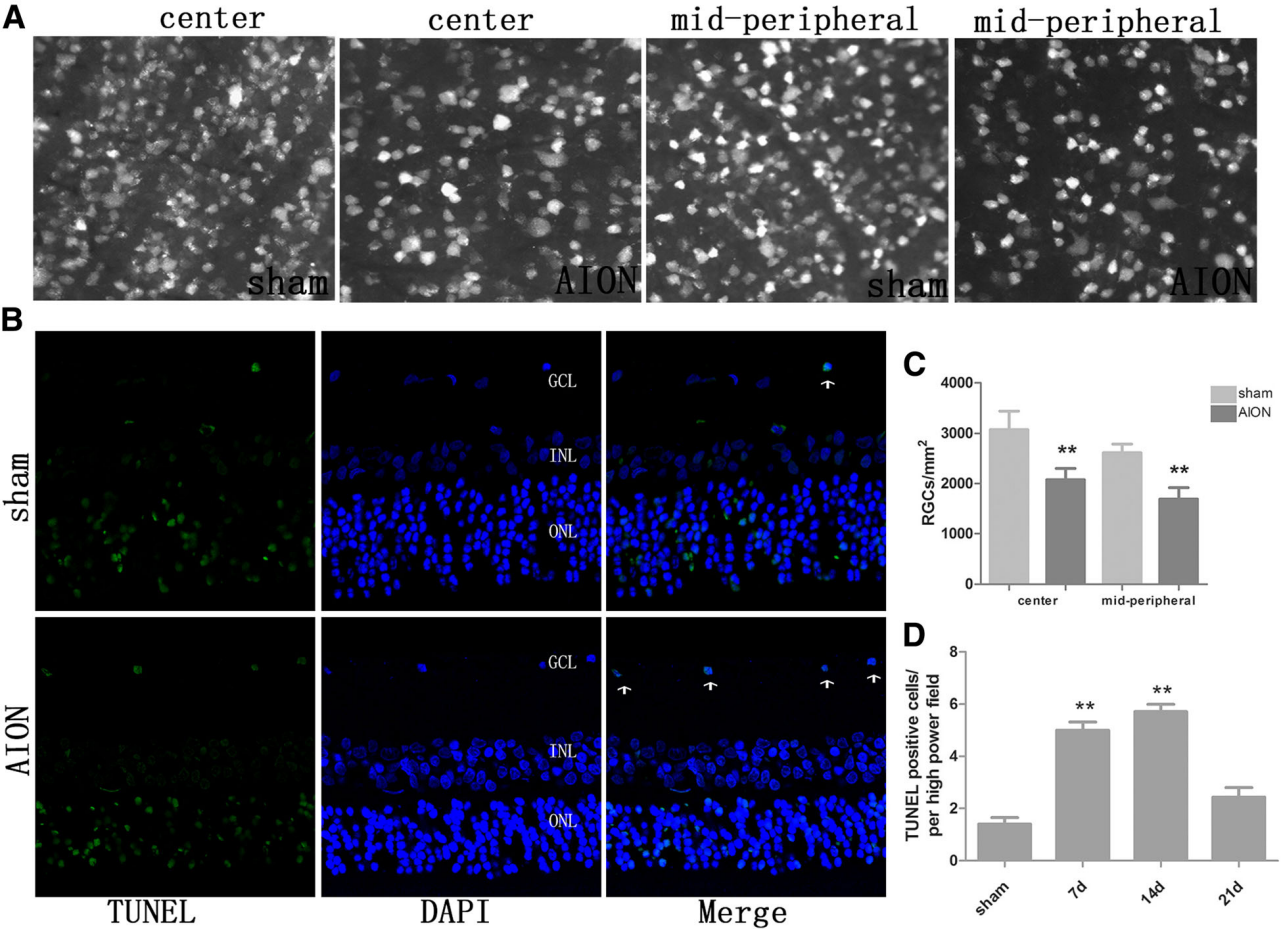
TUNEL-positive cells in the RGC layer and the density of RGC in the retinas. **a**, **c** In the sham group, the densities of RGCs were 3075 ± 298 /mm2 and 2615 ± 138 /mm2 in the central and midperipheral retinas, respectively. And in the photodynamic group, the densities of RGCs were 2078 ± 141 /mm2 and 1691 ± 142 /mm2 in the central and midperipheral retinas, respectively. **p < 0.01 compared with the sham group in the central and midperipheral retinas (*N* = 6 in each group, Bar = 50 um). **b**, **d** At the sham group, bits of TUNEL-positive cells in the RGC layer (1.4 ± 0.55 cells) were detected. At the AION group, the number of TUNEL-positive cells significantly inscreased and reached a peak (5.71 ± 0.76 cells) (**P < 0.01 compared with the sham group, *N* = 6 in each group; field of 200× 200 μm)

The induction of apoptosis was evident by the presence of TUNEL-positive cells in the outer nuclear layer (ONL), inner nuclear layer (INL) and RGC layers of the control eyes. The present study analyzed solely the induction of apoptosis in the RGC layer in treated and sham eyes. On the 1st and the 3rd day following AION induction, negligible induction of apoptosis was noted by the presence of TUNEL- positive cells in GCL, which were not demonstrated in the corresponding figure. On day 7, the number of TUNEL-positive cells significantly increased, and reached a peak (5.71 ± 0.76, *p* < 0.01) by day 14, (Fig. 3b, d) then reduced. At 3 weeks post-AION, the densities of RGCs in the central retinas were 3,075 ± 298 /mm^2^ and 2,078 ± 141 /mm^2^ in the normal and AION eyes, respectively, while in the mid-peripheral retinas the corresponding densities were 2,615 ± 138 /mm^2^ and 1,691 ± 142 /mm^2^, respectively (Fig. 3a, c). The densities of RGCs exhibited significant variation in the treated group compared with the sham group (*N* = 6 in each group, all *p* < 0.01).

### The inflammatory response in retina and ON

**Fig. 4:**
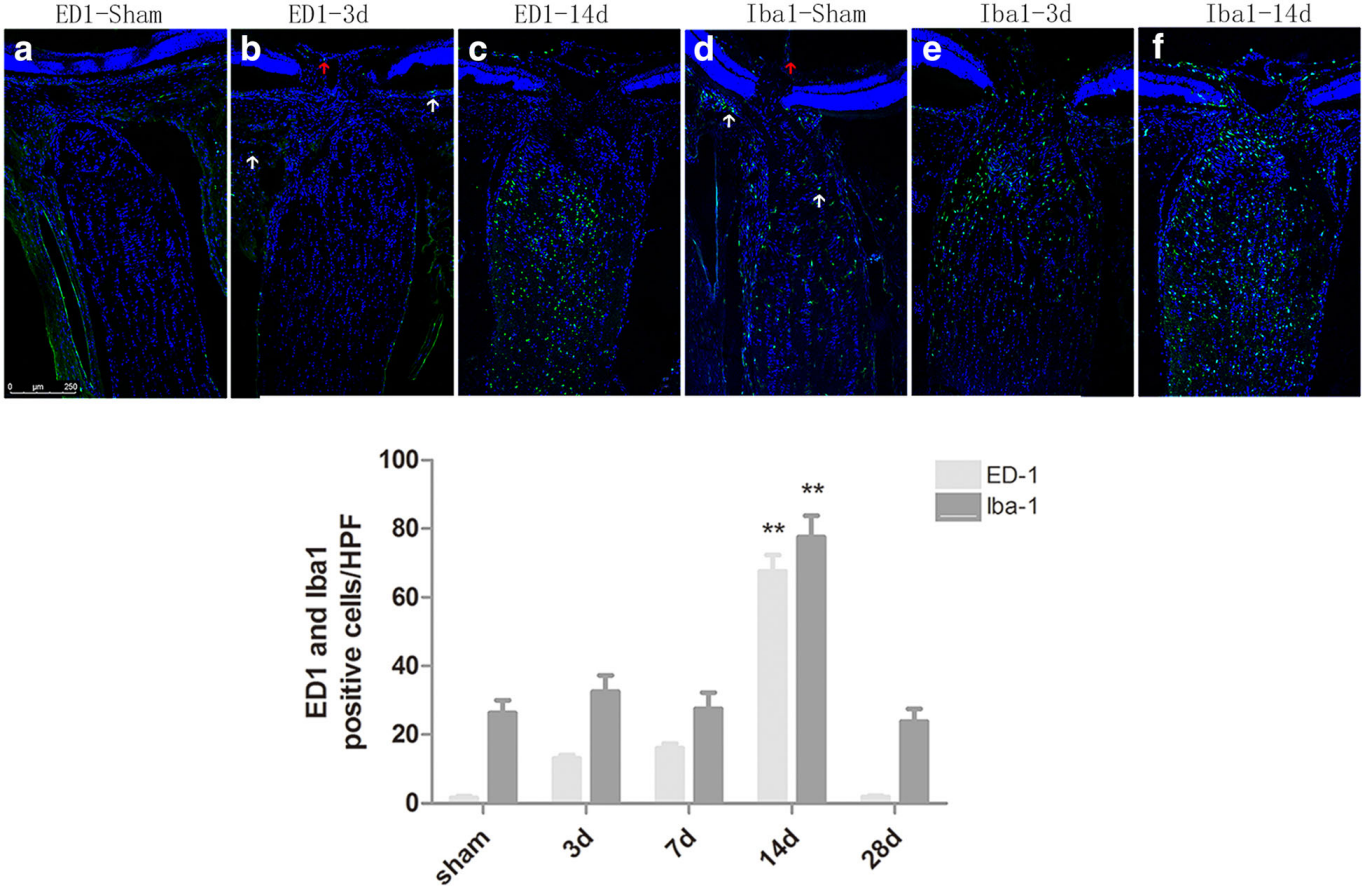
Infiltration of ED-1(+) cells and Iba-1(+) cells both were detected in the ONs post-AION. **a**, **d** At sham group, occasional ED-1(+) cells and distributed Iba-1(+) cells were found in the peripapillary choroid(white arrows) as well as in the anterior ON surrounding a blood vessel(red arrows); **b**, **e** At 3 days post-AION, scattered ED-1(+) cells had a moderate increase especially in the choroid and next to the blood vessels; Iba-1(+) cells had a significat gatheration on the anterior ON; **c**, **f** At 14 days post-AION, ED-1(+) as well as Iba-1(+) cells widely distributed on ON, and both reached the peak (67.5 ± 9.57 cells/HPF and 77.5 ± 12.58 cells/HPF, respectively), **P < 0.01 compared with the sham group (*N* = 6 in each group)

In control ONs, occasional ED-1(+) cells were noted in the peripapillary choroid and in the anterior ON surrounding a blood vessel. By contrast, distributed Iba-1(+) cells were noted in the ON, in the peripapillary choroid, and around the blood vessels (Fig. 4). Following 3 days of treatment, scattered ED-1(+) cells exhibited a moderate increase notably in the choroid and in close proximity to the blood vessels compared with the normal eyes, while Iba-1(+) cells exhibited a significant localization on the anterior ON. On day 14, ED(+) and Iba-1(+) cells were widely distributed on ON, and both reached their maximum number (67.5 ± 9.57 and 77.5 ± 12.58 respectively, *p* < 0.01) during the whole process of the study. Subsequently, ED-1 cells tended to disappear, whereas Iba-1(+) cells exhibited a considerable decrease to a certain number of cells.

## Discussion

In the present study, we aimed to establish an alternative experimental model of laser photoactivation that can be applied for the development of neuroprotective agents against AION. Bernstein et al. were the first to develop a photodynamic model of AION in rats by photoactivating rose Bengal with a 532 nm laser [4]. Since then, this model had been further utilized by various research groups [15–18]. However, the biological half-life of rose Bengal in rats is solely 2 min [19], which indicates that the delay of operation post-injection may weaken the effect of photodynamic treatment on the ON. In the present study, the immediate operation with laser on ON was conducted following injection that can be technically challenging and can lead to variable injured levels of ON. Depending on our clinical experience and previous studies [20, 21], we selected an alternative treatment to induce AION using a dye with easier acquisition and longer half-life.

Preliminary experiments were conducted in order to simplify the experimental operation. Verteporfin was injected via intraperitoneal rather than intravenous injection, but it proved unsuccessful despite the high dose of the photosensitizer and the high energy emission of the laser beam. Considering the fact that the intraperitoneal injection with verteporfin has not been previously reported in rodents [20–22], the present study indicates that the slow accumulation of verteporfin in the retinal circulation may have resulted by the slow peritoneal absorption. Furthermore, the intraperitoneal injection of verteporfin is impractical due to the inability to test the peak plasma concentration and the difficulty of mastering the illumination time. Consequently, the mode of delivery was changed from intraperitoneal injection to intravenous injection. Angiography was carried out with verteporfin at a dose of 6 mg/m^2^ as demonstrated by a previous study [10], in order to test the time duration of the flow of this compound through the choroidal and retinal circulation. Verteporfin was readily detected and was immediately visible in the vascular circulation following the intravenous injection. Following 5 min of injection, verteporfin was visible in both the choroidal and retinal circulation, and after that time period verteporfin appeared to wash out of the vascular circulation. The detection of verteporfin was not possible in the vascular circulation following 10 min of injection. Hence, the operating time was estimated to 10 min post-injection. This time period was sufficient to complete the laser operation and construct an ideal model for AION.

SD-OCT and FFA images of the treated group indicated significant optic nerve edema compared with the sham group on day 1 following the induction of injury. The development of the edema peaked on day 3 and began to decline from day 7. Based on the SD-OCT images, it was noted that certain treated rats had subretinal fluid on day 1. A similar finding has been demonstrated previously in human AION cases [9, 23] and in experimental AION models [24]. The presence of subretinal fluid may promote the death of retinal cells, indicating that the detection of TUNEL-positive cells was possible outside the ganglion cell layer [9]. A wide, non-perfused choroidal vasculature was noted in a certain rat. This unexpected injury may have resulted from individual differences and/or inadvertent operation, and this rat was removed from the study.

TUNEL staining was used to evaluate the apoptotic cell death. Certain parts of TUNEL-positive cells were detected in the outer nuclear layer (ONL) and inner nuclear layer (INL) both in the treated and control eyes, although there was no statistically significant difference (*P* = 0.3) between the two groups. In the treated group, TUNEL-positive cells were demonstrated in the ganglion cell layer by day 7. The percentage of positive cells peaked by day 14 post-ischemia and subsequently declined, which suggested delayed apoptosis following the ON infarct compared to the previous studies [25, 26]. This diversity may have resulted from individual variation and different modeling methods. In the current model, at 3 weeks post-infarct, we could still detect approximately 60% of all RGCs through retrograde labeling of RGCs with FluoroGold. This time period coincided with the seventh day following the peak of apoptosis. Slater et al. [27] demonstrated that at 2–3 weeks post-ischemia, approximately 50% of RGCs were still present. As a result, we speculated that the number of RGCs may be reduced considerably after the 3rd week of treatment until the RGC densities were approximately 30% [8]. The aforementioned findings demonstrated that a prolonged “treatment window” is potentially present in the current model, while the induction of treatment within 7 days post- ischemia may preserve the RGC number. Similarly, such a treatment window may exist in the human AION.

Following injury, extrinsic macrophages were recruited, and resident microglia were activated, at the core of the ischemic ON [8, 28]. This suggests the breakdown of the blood–ON barrier. Activated macrophages exert a dual fuction: They can enhance remyelination and regeneration [29], while they may produce harmful substances –such as pro-inflammatory mediators which can aggravate the neruronal injury. Due to the individual variation and different modeling methods, the current model exhibited a delay of inflammatory response compared to previous studies [5, 28, 30]. Both numbers of ED(+) and Iba-1(+) cells peaked on the 14th day post-infarct and they were subsequently decreased. This suggests additional time for the modulation of the inflammatory process at an early stage.

The functional deficits were demonstrated by gradually decreasing the amplitude of P1 on F-VEP. According to our histological and pathological findings, the decline of vision resulted from the activation of macrophages and the loss of RGCs. We detected subnormal P1 amplitudes on the 7th day. These observations were more profound on the 14th day post-treatment and suggested that cellular degeneration proceeded following the resolution of the ON edema. The visual function was affected by the progressive damage, which is consistent with the findings reported in previous studies [9, 31, 32].

## Conclusion

An AION model was successfully produced as demonstrated by FFA, SD-OCT and F-VEP images. The model can provide the estimation of inflammation by immunofluorescent staining and the estimation of the number of RGCs. The current study provided a steady, technically simple and controlled model, which can be used to examine the potential neuroprotective effects of certain agents and explore the pathological mechanism of AION.

## Abbreviations

AION: Anterior ischemic optic neuropathy
CNV: Choroidal neovascularization
DAPI: 4′,6-diamidino-2-phenylindole
FFA: Fundus Fluorescein angiography
F-VEP: Flash-visual evoked potential
ON: Optic nerve
ONH: Ptic nerve head
PCV: Polypoidal choroidal vasculopathy
RGCs: Retinal ganglion cells
SD-OCT: Spectral domain optical coherence tomography
